# Genome-Wide Analysis of the MADS-Box Gene Family and Expression Pattern Under Abiotic Stresses in *Lilium davidii* var. *unicolor*

**DOI:** 10.3390/ijms27062607

**Published:** 2026-03-12

**Authors:** Xinyi Wang, Yuntao Zhu, Yuwei Nie, Tian Lan, Shuyi Zhang, Yiran Zhao, Jing Wang, Chunli Ma, Hengbin He

**Affiliations:** Beijing Key Laboratory of Ornamental Plants Germplasm Innovation and Molecular Breeding, National Engineering Research Center for Floriculture, Beijing Laboratory of Urban and Rural Ecological Environment, School of Landscape Architecture, Beijing Forestry University, Beijing 100083, China; xinyiwang1@bjfu.edu.cn (X.W.); yuntaozhu1997@gmail.com (Y.Z.); nyw1999@bjfu.edu.cn (Y.N.); lantian@bjfu.edu.cn (T.L.); zsy1221@bjfu.edu.cn (S.Z.); yiranzhao@bjfu.edu.cn (Y.Z.); wangjing010101@bjfu.edu.cn (J.W.); machunli@bjfu.edu.cn (C.M.)

**Keywords:** *Lilium davidii* var. *unicolor*, MADS-box family, phylogenetic analysis, stress response

## Abstract

The MADS-box gene family encodes a critical class of transcription factors that regulate diverse developmental processes in plants. However, its role in abiotic stress responses remains poorly characterized in *Lilium davidii* var. *unicolor* (Lanzhou lily). In this study, we identified 62 *LdMADS* genes in the Lanzhou lily genome, classifying them into 17 Type I and 45 Type II members. Notably, the SOC1 subfamily exhibited a pronounced expansion. These *LdMADS* members were distributed across all twelve chromosomes and displayed considerable structural variation, with some genes harboring exceptionally long introns. Tissue-specific expression profiling revealed that M-type and MIKC* genes were predominantly and specifically expressed in ovaries and anthers, whereas MIKC^C^ members exhibited complex and diverse expression patterns across multiple tissues. The selection of candidate *LdMADS* genes for abiotic stress response was based on their transcript abundance in leaf and root tissues, together with the enrichment of their *cis*-acting elements. The expression of these *LdMADS* genes under drought, heat, and cold stresses was further examined by qRT-PCR. Among them, *LdMADS4* and *LdMADS14* from the SEP subfamily, as well as *LdMADS25* and *LdMADS26* from the SOC1 subfamily, responded to multiple stress conditions. This study provides functional clues for the roles of MADS-box genes in the development and stress responses of Lanzhou Lily.

## 1. Introduction

Transcription factors (TFs) regulate gene expression and perform diverse functions in higher plants [1,2]. They play a major role in plant development, organogenesis, stress responses, and hormone signaling [3,4,5]. Among eukaryotic TFs, the MADS-box family constitutes one of the most extensive groups [6]. All MADS-box genes encode a conserved MADS domain at the N-terminus of the protein, comprising approximately 60 amino acids [7]. MADS-box genes can be classified into two lineages, Type I and Type II, based on their protein domain structure. Type I MADS TFs, also known as M-type TFs, typically contain only one to two exons [8]. They can be further subdivided into three subfamilies (Mα, Mβ, Mγ) based on phylogeny and conserved motifs in their C-terminal regions [9]. In contrast, Type II MADS TFs are generally composed of 7 exons and 6 introns [10]. They feature four characteristic domains known as the MIKC-type TFs. Extending from the N- to the C-terminus, the domain architecture includes the conserved MADS (M) domain, followed by the intervening (I) domain with lower conservation, the keratin-like (K) domain, and finally the variable Carboxy-terminal (C) domain. Distinct I and K domain features form the basis for subdividing them into MIKC^C^ and MIKC* categories [11]. Moreover, MIKC^C^-type MADS TFs can be grouped into 14 distinctive subfamilies: AG/STK, SEP, AGL6, SQUA, DEF/GLO, SOC1, FLC, AGL12, AGL15, SVP, Bsister (GGM13), AGL17, TM8, and OsMADS32-like [12]. Genes within the same subfamily typically exhibit comparable expression profiles, and their encoded proteins often perform closely associated biological functions [13].

The mechanism of DNA binding is fundamental in determining target gene specificity for TFs. MADS-box proteins form dimers that interact with DNA through their conserved MADS domains. In vitro studies have shown that this dimer typically recognizes a 10 bp consensus motif CC(A/T)6GG, referred to as the CArG-box [14]. Furthermore, the evolutionary acquisition of the I and K domains promotes their oligomerization and facilitates interactions with fellow MADS-box proteins, enabling plant MADS-box TFs to form tetramers and achieve greater functional diversification [15].

In higher plants, MADS-box TFs serve as crucial regulators of numerous biological processes, including floral development, nutrient and energy metabolism, signaling and transduction, dormancy release, seed germination, and fruit development [16,17,18]. The primary functions of M-type transcription factors include gametophyte development, embryogenesis, and other reproductive activities [19]. Type II MIKC* members are predominantly expressed in pollen and are essential for pollen germination and maturation in *A. thaliana* [20]. In contrast, MIKC^C^-type genes, which are implicated in virtually every stage of plant development, comprise the majority of MADS-box genes with known functions [21]. Tetramerization of MIKC^C^-type proteins represents a key contributor to flowering diversity among angiosperms. This is exemplified by the well-established ABCDE model of floral organ development, which was formulated after extensive studies clarified the critical role of these complexes in specifying floral organ identity [22,23]. In addition, the MADS-box members *SUPPRESSOR OF OVEREXPRESSION OF CONSTANS1* (*SOC1*), *AGAMOUS-LIKE24* (*AGL24*) [24], *FLOWERING LOCUS C* (*FLC*) [25], and *SHORT VEGETATIVE PHASE* (*SVP*) [26] have also been found to be associated with flowering time and floral transition. Furthermore, *FRUITFULL* (*FUL*) has been implicated in fruit ripening and expansion in diverse species [27,28]. To date, research has increasingly demonstrated the participation of MADS-box TFs in various stress responses. Overexpression of *SlMBP22* in tomato has been demonstrated to enhance drought tolerance by improving water retention, osmolyte accumulation, and antioxidant capacity [29]. In *Oryza sativa*, *OsMADS22*, *OsMADS26*, and *OsMADS55* have been shown to participate in stress responses [30,31]. Notably, the CArG-box motif, recognized by MADS-box proteins, is prevalent in the promoters of numerous defense-related genes. Therefore, elucidating the roles of MADS-box genes under both biotic and abiotic stress provides valuable insights for plant breeding and crop improvement. Despite this potential, the stress-related functions of these genes remain largely unexplored in *Lilium davidii* var. *unicolor*.

*L. davidii* var. *unicolor*, commonly known as the Lanzhou lily, is a perennial herb and a variant of *Lilium davidii.* It predominantly grows in the plateau regions south of Lanzhou City, Gansu Province, China, at elevations between 1800 and 2600 m [32]. The major cultivation area is characterized by a typical temperate continental climate. Precipitation is unevenly distributed and infrequent, often resulting in drought during the growing season. Meanwhile, extreme temperature fluctuations between early spring frost and summer expose plants to both high and low temperature stresses. These abiotic stresses, including drought that inhibits bulb expansion and alters sugar metabolism [32,33], heat that causes growth retardation and developmental delays [34,35], and cold that affects lily plants during the seedling and cut-flower stages [36], have become major limiting factors for the yield and quality of Lanzhou lily. Therefore, elucidating the molecular mechanisms underlying MADS-box gene-mediated abiotic stress responses in Lanzhou lily is urgently needed.

Genetic and breeding studies on the Lanzhou lily have been challenging due to the giant genome size of approximately 36.68 Gb [37]. The completion of its chromosome-level genome assembly in 2024 allowed us to perform the genome-wide characterization of MADS-box genes in Lanzhou lily. Furthermore, we assayed the expression changes of selected members under drought, heat, and cold stress conditions, preliminarily identifying potential key *LdMADS* genes. This study offers a theoretical foundation for enhancing germplasm resources and advancing stress-tolerance studies in Lanzhou lily.

## 2. Results

### 2.1. Identification and Physicochemical Properties of LdMADS Genes

In the genome of *L. davidii* var. *unicolor*, 62 MADS-box genes were identified. The corresponding 62 amino acid sequences are provided in Supplementary Table S1. These 62 genes were systematically named *LdMADS1-LdMADS62* according to their chromosomal locations. The 62 LdMADS proteins range from 110 (LdMADS57) to 423 (LdMADS11) amino acids in length, with molecular weights between 12.449 kDa (LdMADS57) and 48.087 kDa (LdMADS11). Isoelectric point (pI) ranging from 4.92 (LdMADS58) to 10.04 (LdMADS29). Detailed information is provided in Supplementary Table S2.

### 2.2. Phylogenetic Analysis of LdMADS Genes

**Figure 1:**
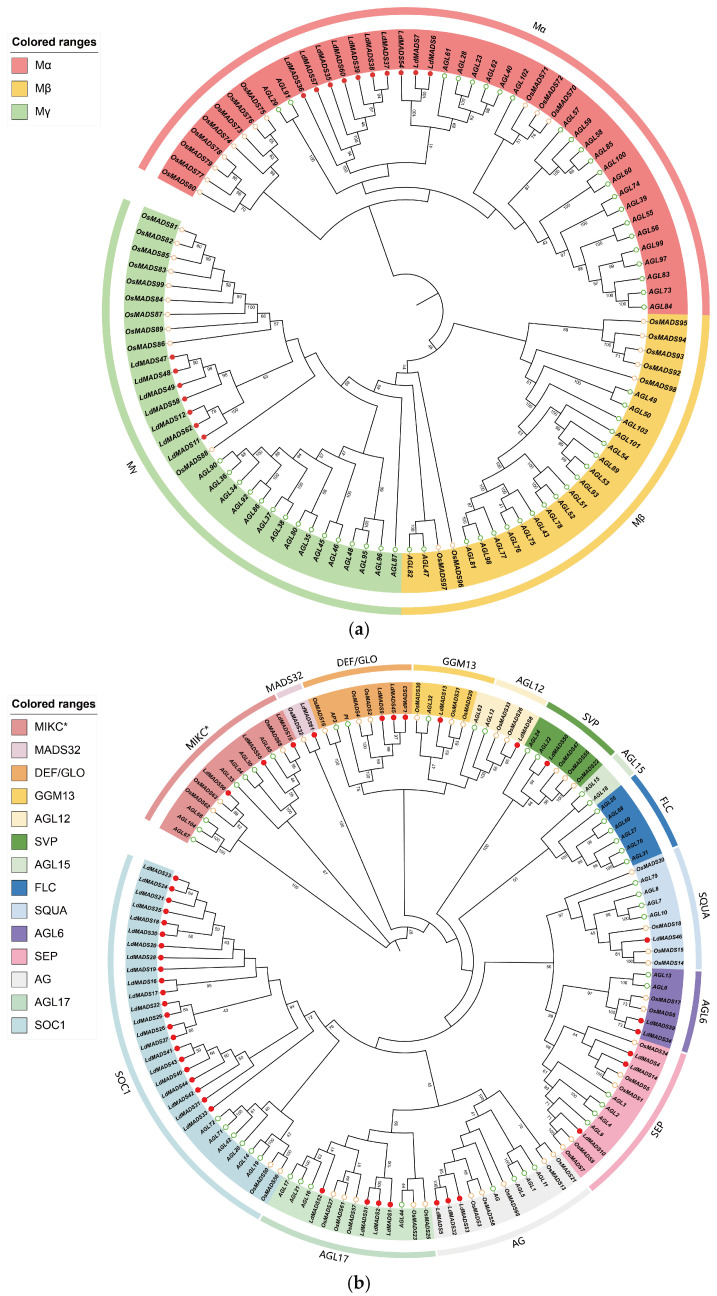
Phylogenetic relationship of MADS-box proteins in Lanzhou Lily, *Arabidopsis*, and rice. LdMADS proteins are indicated by red circles, OsMADS proteins by yellow rings, and AtMADS proteins by green rings. (**a**) Phylogenetic relation of M-type proteins. (**b**) Phylogenetic relation of MIKC proteins.

To examine the phylogenetic relationships of MADS-box genes in Lanzhou Lily, we constructed separate phylogenetic trees for Type I and Type II by aligning their protein sequences with those of *A. thaliana* and *O. sativa* (Figure 1a,b). The phylogenetic results revealed that 17 LdMADS proteins belong to the Type I lineage. These were classified into two subgroups, including 10 Mα and 7 Mγ. Notably, there were no Mβ subgroup members found in the Lanzhou lily genome (Figure 1a). 45 LdMADS proteins were identified as Type II, which included 3 MIKC* and 42 MIKC^C^ members. Furthermore, the MIKC^C^-type LdMADS proteins were distributed into 11 subfamilies, including SEP, AGL6, SQUA, AG, AGL12, SOC1, DEF/GLO, GGM13, AGL17, SVP, and MADS32. No members belonging to the FLC or AGL15 subfamilies were detected (Figure 1b).

Overall, type II proteins in Lanzhou Lily exhibited a quantity similar to that in rice and *Arabidopsis*, whereas the count of Type I proteins was relatively lower. A significant expansion was observed specifically within the SOC1 subfamily, which contains 22 structurally intact SOC1 proteins in Lanzhou Lily.

### 2.3. Conserved Motifs and Gene Structure Analysis of LdMADS Genes

**Figure 2:**
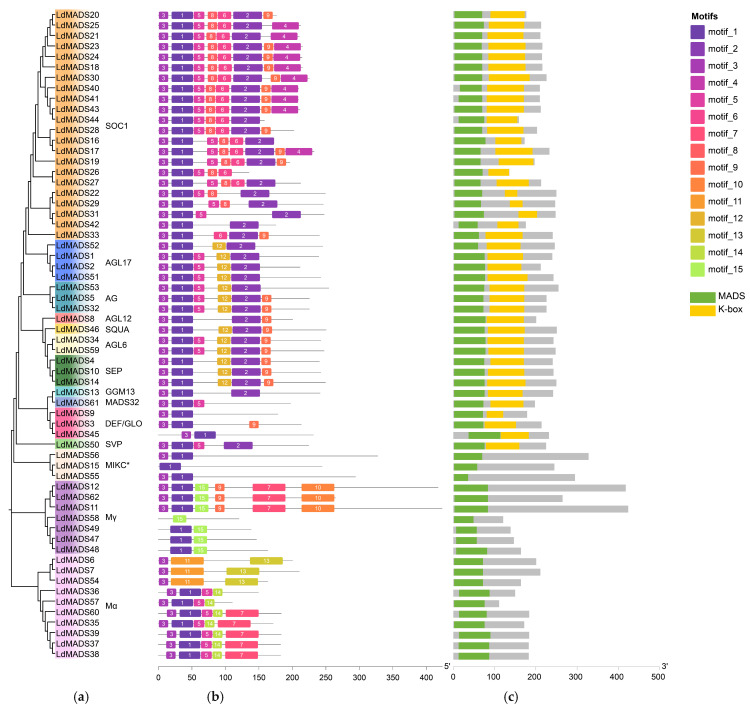
Protein motifs and conserved domains of LdMADS. (**a**) Phylogenetic tree of LdMADS proteins. (**b**) 15 conserved motifs in LdMADS proteins are shown. (**c**) The MADS-domain and K-domain are colored green and yellow, respectively.

The online tool MEME was employed to analyze 62 LdMADS proteins in order to examine the motifs shared among different members and subfamilies, which identified 15 distinct motifs within their amino acid sequences (Figure 2a,b; Table S3). Among these, Motif1 and Motif3 were shared by the majority of LdMADS proteins and together encode the highly conserved M-domain. Motif2, Motif6, Motif9, and Motif12 correspond to K-box and were exclusively found in MIKC proteins (Figure 2b,c). While overall conservation was lower in the C-terminal regions, proteins belonging to the same subfamily tended to possess comparable motif compositions, suggesting a degree of subfamily-specific conservation. These patterns suggest that motif architecture is largely consistent within each subfamily but distinct across different subfamilies, a feature that may reflect their respective functional specializations.

**Figure 3:**
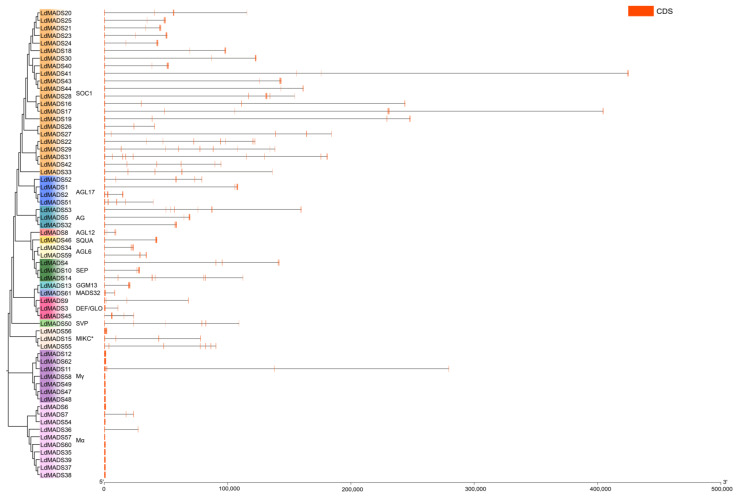
Intron-exon structures of *LdMADS* genes. Intron structures are represented by grey lines; exon structures are represented by red boxes.

To investigate the structural evolution of the 62 *LdMADS* genes, we analyzed their exon-intron organization (Figure 3; Table S2). The analysis revealed considerable variation in gene length and intron number among the *LdMADS* genes, with intron counts ranging from 0 to 10. Specifically, *LdMADS53* and *LdMADS56* contained the highest number of introns, with 10 each. In contrast, 11 genes (*LdMADS6*, *LdMADS12*, *LdMADS35*, *LdMADS37*, *LdMADS38*, *LdMADS39*, *LdMADS48*, *LdMADS57*, *LdMADS58*, *LdMADS60*, and *LdMADS62*) lacked introns entirely and consisted of a single exon. Furthermore, MIKC-type genes generally harbored 3 to 10 introns, whereas M-type genes contained 0 to 3 introns.

Notably, a substantial proportion of *LdMADS* genes were exceptionally long. Thirty genes (48.39%) exceed 50 kb in length and were therefore classified as ultra-long genes [37]. Considerable variation in gene length was also observed even within the same subfamily. Given that the intron count in *LdMADS* genes was comparable to that of other plants, the extraordinary gene length is primarily attributable to extremely long introns. Previous studies indicate that genome expansion in lilies results mainly from the proliferation of transposable elements, which dramatically lengthen intergenic and intronic regions, coupled with contributions from whole-genome duplication and tandem duplication events [38].

### 2.4. Chromosomal Localization and Synteny Analysis

**Figure 4:**
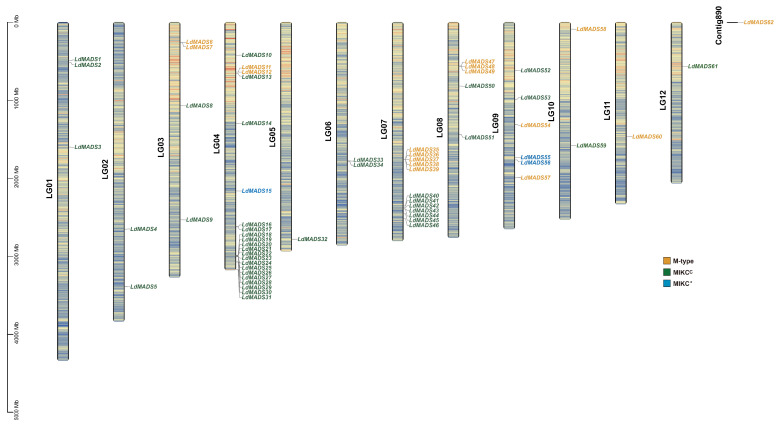
The chromosomal location of *LdMADS*. 61 *LdMADS* genes are distributed across the twelve chromosomes, with one additional gene, *LdMADS62*, located on an unassembled scaffold. Color codes distinguish the gene types, yellow for M-type, blue for MIKC*, and green for MIKC^C^.

To examine genetic divergence and gene duplication events in the MADS-box family, a chromosomal location map was generated from Lanzhou lily genome data. With *LdMADS62* located on an unassembled scaffold, the remaining 61 *LdMADS* members were distributed across all 12 chromosomes, albeit in a highly uneven pattern (Figure 4). Chromosome 4 stands out with the highest count of 22 genes, while chromosome 7 ranks second with 12 genes. Merely one gene was located on each of chromosomes 5, 11, and 12. Members of the Type II SOC1 subfamily are predominantly clustered on chromosomes 4 and 7. Half of the Type I Mα subfamily members are concentrated on chromosome 7.

**Figure 5:**
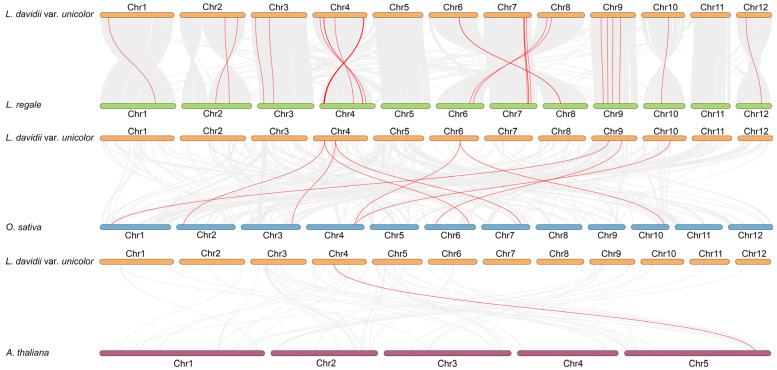
Synteny analysis of the MADS-box genes among *L. davidii* var. *unicolor*, *L. regale*, *O. sativa*, and *A. thaliana*. The gray blocks denote collinear orthologous regions between *L. davidii* var. *unicolor* and other genomes, and the red lines represent syntenic MADS-box gene pairs.

Synteny analysis was performed using MCScanX to compare *LdMADS* genes with their counterparts in *O. sativa*, *Lilium regale*, and *A. thaliana* (Figure 5). A synteny analysis revealed a total of 39 syntenic pairs between the two closely related lily species. Syntenic gene pairs were distributed across all chromosomes with the exception of chromosomes 5 and 11. Chromosome 4 contained the most significant number, accounting for 20 pairs. Conversely, 9 syntenic pairs were detected between Lanzhou lily and *O. sativa*, while only one pair was found between Lanzhou lily and *A. thaliana*. This indicates significant genomic divergence during evolution between monocots (lily, rice) and dicots (*Arabidopsis*). *LdMADS14* exhibited collinear relationships across *L. regale*, *Arabidopsis*, and rice, suggesting its function may be conserved throughout evolution. Indirect evidence for the potential functional conservation of *LdMADS* genes across species was provided by syntenic analysis.

### 2.5. Cis-Acting Elements Analysis of LdMADS Genes

**Figure 6:**
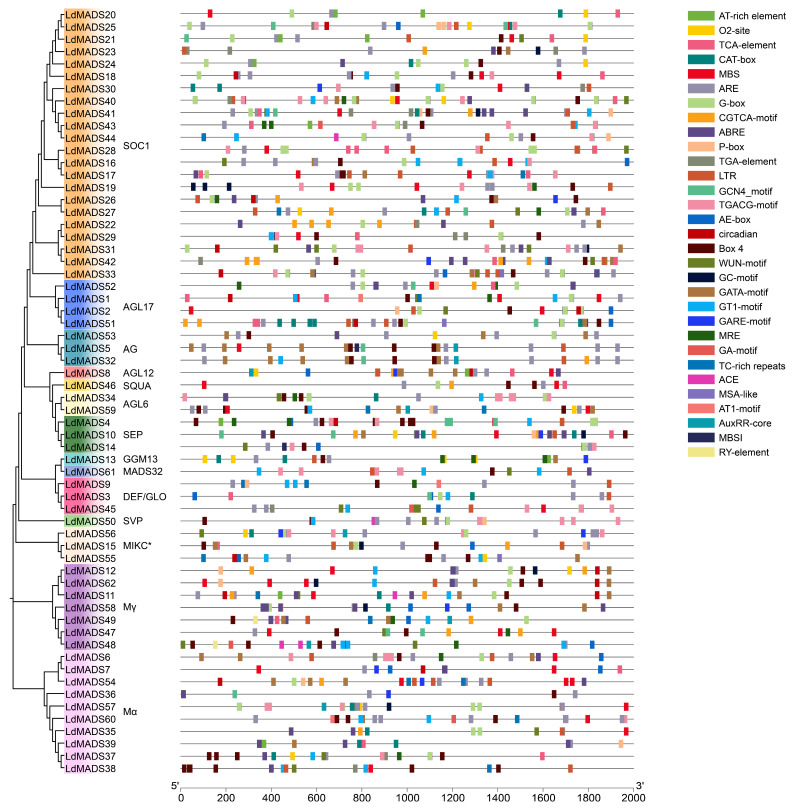
*Cis*-acting elements analysis of *LdMADS* genes. A schematic diagram employs colored squares to represent the types and positional distribution of *cis*-acting elements.

**Figure 7:**
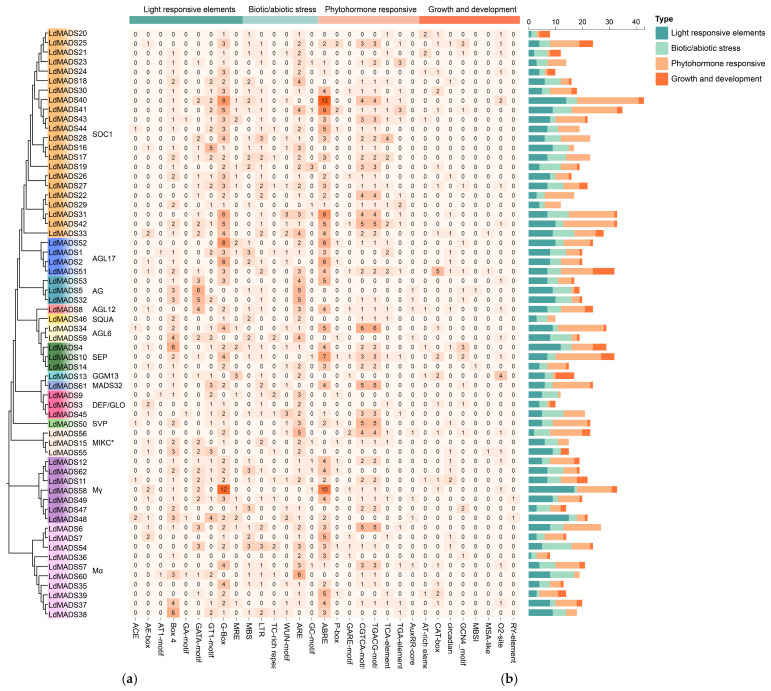
(**a**) The heatmap categorized the promoter elements into four functional classes and enumerated the count of elements within each class for *LdMADS* genes. In the heatmap, the color gradient from pale to orange represents the increasing number of elements. (**b**) The distribution of the four functional element classes across all *LdMADS* promoters was illustrated in a stacked bar chart, with counts per promoter shown.

To further analyze the *cis*-acting elements of *LdMADS* genes, sequences spanning 2000 bp upstream of *LdMADS* genes were extracted. 31 elements with clearly defined biological roles were analyzed using the PlantCARE online database (Figure 6 and Figure 7). These elements could be categorized as light-responsive, stress-responsive, hormone-responsive, and growth regulatory elements. Numerous motifs linked to stress responsiveness and phytohormone regulation were abundant in the promoter region.

The analysis indicated that all candidate genes had at least one stress-related *cis*-acting element, including 49 drought stress response elements (MBS) distributed across 33 *LdMADS* gene promoters and 52 low-temperature response elements (LTR) distributed across 39 *LdMADS* gene promoters (Figure 7). Defense and stress response elements (TC-rich repeats), plant injury response elements (WUN-motifs), and oxidative stress response elements (ARE and GC-motif, etc.) were also identified.

The eight hormone response elements identified can be categorized into five groups, including SA (TCA-element), ABA (ABRE), GA (P-box, GARE-motif), MeJA (CGTCA-motif and TGACG-motif), and auxin (TGA-element and AuxRR-core) response elements. Among them, elements responsive to MeJA were particularly abundant. 197 MeJA-responsive elements were found in the promoters of 44 *LdMADS* genes. Furthermore, it was shown that ABA plays an essential role in the adaptation of nutrient tissues to abiotic stresses, with a total of 178 ABA-responsive elements identified in the promoters of 45 *LdMADS* genes. This evidence lends robust support to the putative function of *LdMADS* genes in mediating abiotic stress adaptation at the transcriptional level.

### 2.6. Tissue-Specific Expression Analysis of LdMADS Genes

**Figure 8:**
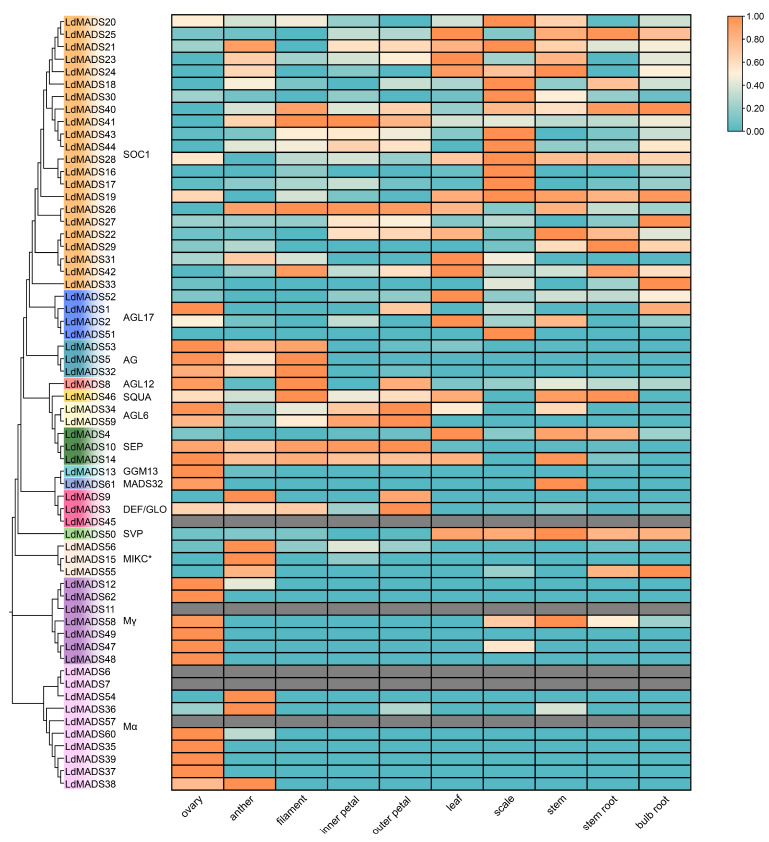
Expression patterns of *LdMADS* genes in different tissues.

Based on public transcriptomic datasets, a heatmap was generated that revealed clear tissue-specific expression profiles and abundance variations for the 62 *LdMADS* genes among ten lily tissues (Figure 8). The five genes that showed no expression across all tissues comprised four Type I genes (*LdMADS6*, *LdMADS7*, *LdMADS11*, *LdMADS57*) and one Type II gene (*LdMADS45*). Ten genes were exclusively expressed in a single tissue. For instance, *LdMADS13*, *LdMADS35*, *LdMADS37*, *LdMADS39*, *LdMADS48*, *LdMADS49*, and *LdMADS62* were expressed solely in the ovary; *LdMADS15* and *LdMADS54* only in the anther; and *LdMADS51* only in the scale. Conversely, nine genes, including *LdMADS14*, *LdMADS19*, *LdMADS20*, *LdMADS26*, *LdMADS28*, *LdMADS40*, *LdMADS41*, *LdMADS42*, and *LdMADS46*, were expressed across all ten tissues.

Type I genes showed more tissue-specific expression than Type II genes. In line with earlier findings that Type I MADS-box genes are crucial for plant reproductive development, the majority of Type I genes were only expressed in the ovary or anther. Their relatively low expression levels imply that they may be active only during specific periods of reproduction. All MIKC*-type genes were discovered to be expressed in anthers, with *LdMADS55* also expressed in stem roots and bulb roots.

In contrast, MIKC^C^ genes displayed broader expression patterns. Most MIKC^C^ genes were detected in two or more tissues. This was particularly evident for the SOC1 subfamily, in which 7 out of the 22 members were expressed across all tissues examined. A predominant expression pattern for these SOC1 subfamily genes was their high expression in the bulb, a specialized storage organ in the lily. In addition to *SOC1*, scales also exhibit significant expression of homologs of *SVP* (*LdMADS50*) and *FUL* (*LdMADS46*). Members of the same subfamily typically exhibit comparable expression profiles. This is exemplified by the three AG subfamily genes, which show pronounced and specific expression in anthers, filaments, and ovaries. Two of the three genes of the SEP subfamily (*LdMADS10* and *LdMADS14*) were strongly expressed in all floral organs, while two genes (*LdMADS14* and *LdMADS4*) exhibited high transcript levels in leaves. The expression patterns of *LdMADS* genes show clear subfamily specificity and are consistent with earlier research on their roles in floral organ development.

Analysis of the tissue expression data showed that members of the *LdMADS* gene family exhibit complex and distinct expression patterns, suggesting a potential association with their functional diversification.

### 2.7. Expression Patterns of LdMADS Genes Under Stress Conditions

**Figure 9:**
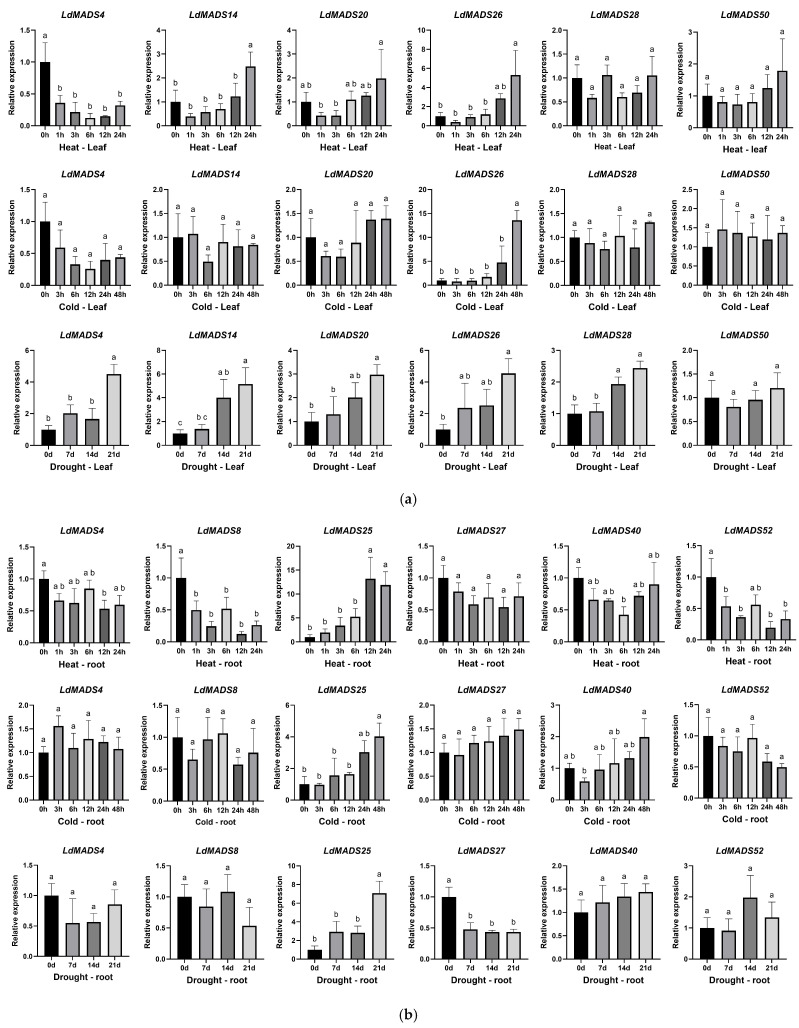
Expression patterns of selected *LdMADS* genes under stress conditions. Plants were treated with heat (37 °C), cold (4 °C), or drought stress. Following this, relative expression levels were measured at a series of time points. Different lowercase letters (a, b, and c) above the bars indicate statistically significant differences at *p* < 0.05 (one-way ANOVA with Tukey’s multiple comparisons test). (**a**) Expression in leaf tissue. (**b**) Expression in root tissue.

To investigate the potential role of the *LdMADS* gene family in stress response, we combined analyses of root and leaf expression levels and promoter element distributions with prior functional studies of MADS-box genes. Based on this integrated analysis, six candidate genes were selected for further study. In leaves, these were *LdMADS4*/*14* (*SEP2*), *LdMADS20*/*26*/*28* (*SOC1*), and *LdMADS50* (*SVP*). In roots, these were *LdMADS4* (*SEP2*), *LdMADS8* (*AGL12*), *LdMADS25*/*27*/*40* (*SOC1*), and *LdMADS52* (*AGL16*). Their expression patterns under cold (4 °C), heat (37 °C), and drought stress were then analyzed by qRT-PCR (Figure 9).

Examination of the six genes expressed in leaves revealed that all but *LdMADS50* responded to at least one stress, showing differences in expression patterns. Notably, under low-temperature conditions, only *LdMADS26* (a homolog of *SOC1*) was significantly induced, reaching more than ten times its pre-treatment level after 48 h of treatment. This response was consistent with the presence of an LTR motif in its promoter. In contrast, the expression changes of other genes, including the *SVP* homolog *LdMADS50*, did not show a significant change at low temperature. Under high-temperature stress, *LdMADS4* expression decreased, reaching its lowest level at 6 h before a slight rebound, while *LdMADS14*/*20*/*26* exhibited a delayed up-regulation. All screened genes except *LdMADS50* responded to drought. *LdMADS4*/*14*/*20*/*26*/*28* demonstrated sustained transcriptional upregulation, indicating an ongoing response process.

Detection of the six genes expressed in roots revealed that two *SOC1* members, *LdMADS25* and *LdMADS40,* were up-regulated in response to low temperature. *LdMADS40* showed a slight decrease in expression after 3 h of low-temperature treatment, exhibiting a delayed response. Under high-temperature stress, the expression patterns of different *LdMADS* genes varied greatly. *LdMADS4*/*8*/*52* expression decreased in general and reached its lowest at 12 h, while *LdMADS25* was strongly upregulated, with its peak expression at 12 h. This common time point may indicate that 12 h is a critical period for responding to high-temperature stress. *LdMADS40* also responded to high temperature in roots, with expression first decreasing and then increasing. *LdMADS25* and *LdMADS40* responded to drought stress, but showed opposite expression patterns.

Based on the qRT-PCR results, we observed that SOC1 subfamily members exhibited distinct stress-responsive expression patterns. *LdMADS25* (roots) and *LdMADS26* (leaves) were strongly upregulated under all three stress conditions, suggesting they may function as positive regulators involved in multiple stress responses. Notably, SOC1 subfamily members were the only genes among our selected candidates that responded to cold stress in both roots (*LdMADS25/40*) and leaves (*LdMADS26*). In contrast, the *SEP2* homologs *LdMADS4* and *LdMADS14* were primarily responsive to high temperature and drought, though their specific expression patterns differed. This suggests that the expanded *SOC1* members in Lanzhou lily may partly function as positive regulators in mediating various stress responses.

## 3. Discussion

In this study, 62 MADS-box genes were identified in Lanzhou lily. In accordance with their chromosomal locations, these genes were designated as *LdMADS1* to *LdMADS62*. The *LdMADS* members were categorized into two major groups: Type I (Mα 10 genes, Mγ 7 genes) and Type II MIKC (MIKC* 3 genes, MIKC^C^ 42 genes). The MIKC^C^ genes were subsequently assigned to 11 distinct subfamilies. The number of MADS-box genes varies significantly across species. For instance, *O. sativa* has 75 [39], *Sorghum bicolor* has 65 [40], and *A. thaliana* has 107 [8]. Although the genome of Lanzhou lily is markedly larger than that of other species, the total number of *LdMADS* genes is not correspondingly greater. Further analysis revealed a relatively low number of M-type genes and the absence of members from the Mβ subgroup. Unlike the MIKC genes, Type I genes are typically characterized by shorter lengths and have undergone more rapid birth and death during evolution [9,41]. They often expand through small-scale duplication events, making them more prone to silencing or loss in the genome [42]. Nevertheless, a subset of Type I genes plays crucial roles in key reproductive developmental pathways, such as *AGL23*, *AGL61*, *AGL62*, and *AGL80* [43,44,45,46]. In *L. davidii* var. *unicolor*, multiple homologs of these genes were identified among its Type I members and were found to be specifically expressed in reproductive tissues. This suggests potential conservation of the structure and function of Type I genes across angiosperms. In contrast, Type II MADS-box genes are preferentially retained following whole-genome or large-scale duplication events. This differential retention may explain why the copy number of Type I MADS-box genes exhibits greater variation than that of Type II genes across species [21].

Expansion of specific MADS-box subfamilies has been documented in various plants. For instance, the SOC1 subfamily of *Eucalyptus grandis* has undergone significant expansion. The subfunctionalization of *SOC1* enables adaptation to variable ecological environments spanning the tropics to temperate zones, thereby meeting different needs in flowering regulation [47,48]. In addition, the expansion of FLC and SEP subfamilies has also been observed in some Asteraceae species, such as *Taraxacum kok-saghyz* [49] and *Taraxacum officinale* [50]. In Lanzhou Lily, the SOC1 subfamily has undergone a remarkable expansion, with 22 members identified, accounting for nearly half of all its MIKC-type genes. This proportion is substantially higher than that reported in other angiosperms. The expansion pattern of the SOC1 subfamily in Lanzhou Lily appears complex. Beyond two pairs of tandem duplicate genes (*LdMADS20*/*LdMADS21* and *LdMADS25*/*LdMADS26*), most *SOC1* members form local clusters on chromosomes 4 and 7. These genes are physically proximate yet interspersed by a few other genes, consistent with the characteristics of proximal duplication [51,52]. Compared to other duplication modes, tandem and proximal duplicates often undergo highly selective pressure, exhibit more compact gene structures and broader expression patterns, and serve as a key genetic reservoir for evolving novel functions [51]. Transcriptomic data reveal that the expanded *SOC1* genes exhibit broad and diverse expression patterns across the 10 tissues examined in Lanzhou Lily. Most are highly expressed in the bulb, a specialized storage organ, suggesting potential subfunctionalization or neofunctionalization within this subfamily [53]. A particularly notable observation is the absence of FLC subfamily members in the Lanzhou lily genome. In *Arabidopsis*, *FLC* is a master regulator of vernalization, which is a low-temperature requirement for flowering that lilies also exhibit [54]. This suggests that functional diversification among the SOC1 subfamily in lilies may compensate for or replace the regulatory role of *FLC* in the vernalization pathway. Consistent with this hypothesis, *SOC1* in Lanzhou lily is highly expressed in overwintering organ bulbs and responds to cold stress. These expanded *SOC1* homologs may therefore not only function as integrators of flowering signals but also participate in *Lilium*-specific developmental processes, such as bulb formation, dormancy release, and vernalization [55].

The evolution of plant MADS-box genes involves the loss and gain of introns [56]. Typically, Type II MIKC members contain more introns than Type I members. Analysis of the gene structures revealed that Type II members contained an average of 6.4 introns, whereas most Type I genes lack introns entirely, except for a few exceptions. The intron number in *LdMADS* genes is similar to that observed in *Arabidopsis*, rice, and other plants. However, Lanzhou lily contains a gigantic genome, and this is reflected in gene length. Previous genomic analysis showed that 33.88% of all annotated genes in Lanzhou lily exceed 50 kb in length and are defined as ultra-long genes [37]. Strikingly, this proportion rises to 48.39% within the *LdMADS* gene family. Despite their ultra-long lengths, the exon structures of these *LdMADS* genes remain conserved, with average number and length comparable to those found in other plants. Thus, the extensive lengthening of these genes is primarily due to extreme intron elongation. Previous studies indicate that the dramatic increase in lily genome size is largely associated with bursts of transposable elements insertions, which accumulate abundantly in intergenic regions and introns [37]. The preferential accumulation of transposable element sequences in MADS-box introns may have introduced new regulatory elements, potentially adding complexity to the transcriptional regulation of *LdMADS* genes.

Tissue-specific expression analysis revealed that *LdMADS* genes exhibit highly divergent and specific expression patterns, which are closely linked to their functional diversity. M-type genes were expressed mainly at low abundance in the ovary and anther, consistent with their conserved function in plant reproductive development. Conversely, MIKCC genes showed a broader expression profile. Of particular note was the SOC1 subfamily, of which 7 of 22 members were detected across all tissues tested. Similarly, homologs of *SVP* (*LdMADS50*) and *FUL* (*LdMADS46*) were also highly expressed in scales, suggesting their potential common role in lily-specific biological processes, including dormancy release and environmental adaptation. In addition, three AG subfamily genes showed pronounced and specific expression in the anther, filament, and ovary. *AG* is a representative gene that characterizes the floral organs of class C and is studied to be expressed in stamens and carpels [57]. Two SEP subfamily genes (*LdMADS10* and *LdMADS14*) were highly expressed in all five floral organ tissues. The specialization of floral organ features depends on SEP subfamily members, which are usually defined as class E genes [58]. In conclusion, tissue expression profiling not only confirmed the important function of the MADS-box family in floral development but also suggested its possible role in bulb formation and in adaptation to environmental stress.

Our investigation of promoter regions identified a prevalent abundance of abiotic stress-responsive *cis*-elements within the *LdMADS* promoters. Elements associated with hormone signaling, drought, and low-temperature stress were frequently present. Consistent with this putative role in stress adaptation, MADS-box genes are known regulators of abiotic stress responses in other species. As evidenced by studies in *A. thaliana*, *AGL16* acts as a negative regulator of drought resistance by modulating stomatal density and ABA accumulation, and by directly regulating *CYP707A3*, *AAO3*, and *SDD1* [59]. In *Capsicum annuum*, a SEP subfamily member, *CaMADS*, positively regulates abiotic stress responses and is induced by multiple stresses and hormones [60]. Based on prior studies, tissue expression profiles, and promoter element analyses, we validated the expression patterns of selected *LdMADS* genes under drought, cold, and heat stress.

*LdMADS14*, a homolog of *SEP2*, was also found to be significantly up-regulated in leaves under both drought and heat stresses, while *LdMADS4* responded to various stresses in both leaves and roots. *SOC1* homologs *LdMADS25* and *LdMADS26* were strongly induced in response to all three stresses in roots and leaves, respectively. The upregulation of *LdMADS25* in leaves under cold stress, together with the responsiveness of *LdMADS26* and *LdMADS40* in roots, further supports the involvement of the expanded SOC1 subfamily in cold response. Notably, although LTR elements were identified in the promoters of multiple candidate genes, only *SOC1* homologs exhibited significant upregulation under cold stress. This may suggest that the cold response of MADS-box genes involves complex transcriptional regulation beyond the mere presence of individual *cis*-elements. Conversely, *LdMADS8* (an *AGL12* homolog) and *LdMADS52* (an *AGL16* homolog) were down-regulated in roots under heat stress. Collectively, the results suggest that specific *LdMADS* genes, especially *SOC1* and *SEP* homologs, may be crucial for enhancing stress tolerance as regulators of multiple stresses in Lanzhou lily, while their precise molecular mechanisms remain to be further investigated.

## 4. Materials and Methods

### 4.1. Identification of LdMADS Gene Family Members

The genomic data of Lanzhou lily were acquired from the China National GeneBank Database (https://ftp.cngb.org/pub/CNSA/data5/CNP0005511/CNS1065710/CNA0139751/, accessed on 15 January 2025) [37]. Identification of MADS-box genes within the Lanzhou lily genome was conducted employing HMMER-3.0 software [61]. Corresponding hidden Markov model (HMM) profiles for the SRF (Type I) domain (PF00319) and the MEF2-like (Type II) domain (PF09047) were obtained from the Pfam database [62]. In addition, MADS-box proteins from *Arabidopsis* and rice served as query sequences for BlastP searches against the predicted proteins. The TAIR website (http://www.arabidopsis.org/) provided the *Arabidopsis* MADS-box family database [8], while the Rice Genome Annotation Project (http://rice.plantbiology.msu.edu/) provided the rice MADS-box family database [39]. The presence of the conserved domain was then confirmed for each candidate protein sequence using the SMART database (https://smart.embl.de). Redundant sequences and those lacking a complete MADS domain were removed from the final dataset. Additionally, we analyzed the physicochemical properties of the identified LdMADS proteins using the Protein Parameter Calc tool in TBtools [63].

### 4.2. Phylogenetic Analysis of LdMADS Proteins

Using amino acid sequences of the 62 *LdMADS* genes and known MADS-box sequences from *Arabidopsis* and rice, a phylogenetic tree was constructed to confirm the identification. Sequence alignment was carried out with the Clustal W algorithm in MEGA11 software. For statistical support, the Neighbour-Joining (NJ) method was applied with 1000 bootstrap replicates. The iTOL online tool (https://itol.embl.de) was used to visualize and annotate the resultant tree.

### 4.3. Analysis of Protein Conserved Motifs, Domains, and Gene Structures

Conserved motifs in the LdMADS proteins were detected using the online MEME suite (https://meme-suite.org/meme/, accessed on 24 June 2025), with a maximum of 15 motifs configured while retaining all other default parameters. Domain prediction was performed via analysis of the MADS-box protein sequences using the NCBI Conserved Domain Database (CDD). Moreover, the results of the conserved motif analysis, domain predictions, and the genome annotation file (gff3) were merged and visualized using the Gene Structure View function in TBtools.

### 4.4. Chromosomal Localization and Synteny Analysis of LdMADS Genes

The chromosomal locations of the *LdMADS* genes were mapped and visualized using the Gene Location Visualize function in TBtools (version 2.423). Synteny analysis between the *LdMADS* genes and the MADS-box members from rice, *L. regale*, *and Arabidopsis* was analyzed using the One Step MCScanX-Super Fast tool.

### 4.5. Cis-Acting Element Analysis of LdMADS Genes

The promoter region 2000 bp upstream of 62 *LdMADS* genes was extracted using the Gtf/Gff3 Sequences Extract tool in TBtools. To complete the prediction and analysis of *cis*-acting elements, the *LdMADS* gene promoter sequences were uploaded to the PlantCARE database (https://bioinformatics.psb.ugent.be/webtools/plantcare/html/, accessed on 25 June 2025). The resulting element profiles were subsequently counted and classified accordingly.

### 4.6. Tissue-Specific Expression Profiling of LdMADS Genes

To investigate their potential functions, the expression profiles of *LdMADS* genes were analyzed across ten tissues. Transcriptomic data for anther, filament, inner tepal, outer tepal, leaf, ovary, stem, stem-derived root, and basal root were obtained from the RNA-seq dataset published by Xu et al. (BioProject ID: CNP0005511) [37]. Data for the scale tissue were sourced from a separate RNA-seq study by Qi et al. (BioProject ID: PRJNA705898) [64].

### 4.7. Plant Materials and Treatments

Planting was conducted at the agricultural experiment base of Beijing Forestry University (116.34 E, 40.01 N). Uniformly sized bulbs were transplanted into pots (16 cm in diameter) with three bulbs per pot, constituting one biological replicate. Plants were grown for 30 days, after which healthy individuals of consistent size and vigor were selected for the stress treatment.

For the drought stress treatment, potted plants were placed in trays, and each pot was irrigated with 500 mL of tap water until the soil reached full water-holding capacity. The collected osmotic water from the trays was reapplied to ensure a balanced amount. All plants were then transferred to a growth chamber at 25 °C, 60% relative humidity, and a 16/8 h light/dark photoperiod. Watering was subsequently withheld completely. Soil water content was measured, and samples were collected from the upper leaves and stem roots at 0, 7, 14, and 21 days after the start of the treatment.

For the heat stress treatment, plants were placed at 37 °C under identical humidity and photoperiod conditions. Sampling was performed at 0, 1, 3, 6, 12, and 24 h after treatment initiation. For cold stress treatment, plants were placed at 4 °C. Samples were acquired at 0, 3, 6, 12, 24, and 48 h.

Each treatment included three biological replicates. Plant samples were encased in aluminum foil, rapidly frozen in liquid nitrogen, and subsequently preserved at −80 °C for subsequent analysis and RNA extraction.

### 4.8. qRT-PCR Analysis of the LdMADS Genes

Total RNA was extracted from the samples using the EASY Spin Plus RNA Kit (Aldlab, Beijing, China). Following evaluation of both quantity and quality, sufficient amounts of cDNA were synthesized using a reverse transcription kit (TOYOBO, Shanghai, China). Quantitative real-time PCR (qRT-PCR) reactions were conducted on a CFX Connect instrument (Bio-Rad, Shanghai, China) with SYBR Green Pro Taq HS (Accurate Biology, Changsha, China). The *LdActin* gene was utilized as a control to standardize expression levels, and primer sequences were presented in Table S4. The relative gene expression of *LdMADS* genes was calculated using the 2^−ΔΔCt^ method.

## 5. Conclusions

This study presented the first genome-wide identification of the MADS-box transcription factor family in Lanzhou lily, a species with a giant genome. Two key genomic features were identified: a significant expansion of the SOC1 subfamily and the prevalence of ultra-long genes driven by intron elongation. Combined with a tissue-specific expression heatmap, we propose that these expanded genes may not only regulate flowering time but also perform essential functions in lily-specific processes, including bulb development, dormancy regulation, and stress responses. This shows the functional diversification of this subfamily in the Lanzhou lily. The results of the stress response analyses indicated that some *LdMADS* members, particularly *LdMADS4*, *LdMADS14*, *LdMADS25*, and *LdMADS26*, are likely involved in abiotic stress adaptation. Collectively, these findings supply a genetic resource and potential candidate genes for further research on stress tolerance in Lanzhou lily.

## Data Availability

Data are contained within the article and Supplementary Materials.

## Supplementary Materials

The following supporting information can be downloaded at: https://www.mdpi.com/article/10.3390/ijms27062607/s1.

## References

[1] Riechmann J.L., Ratcliffe O.J. (2000). A genomic perspective on plant transcription factors. Curr. Opin. Plant Biol..

[2] Singh K., Foley R.C., Onate-Sanchez L. (2002). Transcription factors in plant defense and stress responses. Curr. Opin. Plant Biol..

[3] Messenguy F., Dubois E. (2003). Role of MADS box proteins and their cofactors in combinatorial control of gene expression and cell development. Gene.

[4] Gahlaut V., Jaiswal V., Kumar A., Gupta P. (2016). Transcription factors involved in drought tolerance and their possible role in developing drought tolerant cultivars with emphasis on wheat (*Triticum aestivum* L.). Theor. Appl. Genet..

[5] Dong X., Deng H., Ma W., Zhou Q., Liu Z. (2021). Genome-wide identification of the MADS-box transcription factor family in autotetraploid cultivated alfalfa (*Medicago sativa* L.) and expression analysis under abiotic stress. BMC Genom..

[6] Riechmann J.L., Heard J., Martin G., Reuber L., Jiang C., Keddie J., Adam L., Pineda O., Ratcliffe O.J., Samaha R.R. (2000). *Arabidopsis* transcription factors: Genome-wide comparative analysis among eukaryotes. Science.

[7] de Folter S., Angenent G.C. (2006). *trans* meets *cis* in MADS science. Trends Plant Sci..

[8] Parenicová L., de Folter S., Kieffer M., Horner D., Favalli C., Busscher J., Cook H., Ingram R., Kater M., Davies B. (2003). Molecular and phylogenetic analyses of the complete MADS-box transcription factor family in *Arabidopsis*: New openings to the MADS world. Plant Cell.

[9] De Bodt S., Raes J., Florquin K., Rombauts S., Rouzé P., Theissen G., Van de Peer Y. (2003). Genomewide structural annotation and evolutionary analysis of the type I MADS-box genes in plants. J. Mol. Evol..

[10] Lai X., Vega-Léon R., Hugouvieux V., Blanc-Mathieu R., van der Wal F., Lucas J., Silva C., Jourdain A., Muino J., Nanao M. (2021). The intervening domain is required for DNA-binding and functional identity of plant MADS transcription factors. Nat. Commun..

[11] Henschel K., Kofuji R., Hasebe M., Saedler H., Münster T., Theißen G. (2002). Two Ancient Classes of MIKC-type MADS-box Genes are Present in the Moss Physcomitrella patens. Mol. Biol. Evol..

[12] Smaczniak C., Immink R., Muiño J., Blanvillain R., Busscher M., Busscher-Lange J., Dinh Q., Liu S., Westphal A., Boeren S. (2012). Characterization of MADS-domain transcription factor complexes in *Arabidopsis* flower development. Proc. Natl. Acad. Sci. USA.

[13] Zhao W., Zhang L., Xu Z., Fu L., Pang H., Ma Y., Min D. (2021). Genome-Wide Analysis of MADS-Box Genes in Foxtail Millet (*Setaria italica* L.) and Functional Assessment of the Role of *SiMADS51* in the Drought Stress Response. Front. Plant Sci..

[14] Tilly J.J., Allen D.W., Jack T. (1998). The CArG boxes in the promoter of the *Arabidopsis* floral organ identity gene *APETALA3* mediate diverse regulatory effects. Development.

[15] Hugouvieux V., Silva C.S., Jourdain A., Stigliani A., Charras Q., Conn V., Conn S.J., Carles C.C., Parcy F., Zubieta C. (2018). Tetramerization of MADS family transcription factors SEPALLATA3 and AGAMOUS is required for floral meristem determinacy in *Arabidopsis*. Nucleic Acids Res..

[16] Bowman J., Moyroud E. (2024). Reflections on the ABC model of flower development. Plant Cell.

[17] Guo L., Luo X., Li M., Joldersma D., Plunkert M., Liu Z. (2022). Mechanism of fertilization-induced auxin synthesis in the endosperm for seed and fruit development. Nat. Commun..

[18] Niu Q., Li J., Cai D., Qian M., Jia H., Bai S., Hussain S., Liu G., Teng Y., Zheng X. (2016). Dormancy-associated MADS-box genes and microRNAs jointly control dormancy transition in pear (*Pyrus pyrifolia* white pear group) flower bud. J. Exp. Bot..

[19] Hoffmann T., Shi X., Hsu C.Y., Brown A., Knight Q., Courtney L.S., Mukarram R.J., Wang D. (2022). The identification of type I MADS box genes as the upstream activators of an endosperm-specific invertase inhibitor in *Arabidopsis*. BMC Plant Biol..

[20] Zhang L., Ma F., Duan G., Ju Y., Yu T., Zhang Q., Sodmergen S. (2024). MIKC*-type MADS transcription factors control JINGUBANG expression and the degree of pollen dormancy in *Arabidopsis*. Plant Physiol..

[21] Gramzow L., Theissen G. (2010). A hitchhiker’s guide to the MADS world of plants. Genome Biol..

[22] Theissen G. (2001). Development of floral organ identity: Stories from the MADS house. Curr. Opin. Plant Biol..

[23] Silva C., Puranik S., Round A., Brennich M., Jourdain A., Parcy F., Hugouvieux V., Zubieta C. (2016). Evolution of the Plant Reproduction Master Regulators LFY and the MADS Transcription Factors: The Role of Protein Structure in the Evolutionary Development of the Flower. Front. Plant Sci..

[24] Liu C., Chen H., Er H., Soo H., Kumar P., Han J., Liou Y., Yu H. (2008). Direct interaction of *AGL24* and *SOC1* integrates flowering signals in *Arabidopsis*. Development.

[25] Lee H., Park H., Lee K., Lee J., Kim J. (2023). Two *Arabidopsis* Splicing Factors, U2AF65a and U2AF65b, Differentially Control Flowering Time by Modulating the Expression or Alternative Splicing of a Subset of *FLC* Upstream Regulators. Plants.

[26] Lee J.H., Yoo S.J., Park S.H., Hwang I., Lee J.S., Ahn J.H. (2007). Role of *SVP* in the control of flowering time by ambient temperature in *Arabidopsis*. Genes Dev..

[27] Eriksson E.M., Bovy A., Manning K., Harrison L., Andrews J., De Silva J., Tucker G.A., Seymour G.B. (2004). Effect of the Colorless non-ripening mutation on cell wall biochemistry and gene expression during tomato fruit development and ripening. Plant Physiol..

[28] Tang L., He Y., Liu B., Liu M., Xu Y., Zhang J., Kong W., An L., Hu K., Garcia-Mas J. (2025). *CmFUL1* was potentially involved in fruit elongation in melon. Hortic. Res..

[29] Li F., Chen X., Zhou S., Xie Q., Wang Y., Xiang X., Hu Z., Chen G. (2020). Overexpression of *SlMBP22* in Tomato Affects Plant Growth and Enhances Tolerance to Drought Stress. Plant Sci..

[30] Lee S., Choi S., An G. (2008). Rice SVP-group MADS-box proteins, OsMADS22 and OsMADS55, are negative regulators of brassinosteroid responses. Plant J..

[31] Khong G., Pati P., Richaud F., Parizot B., Bidzinski P., Mai C., Bès M., Bourrié I., Meynard D., Beeckman T. (2015). OsMADS26 Negatively Regulates Resistance to Pathogens and Drought Tolerance in Rice. Plant Physiol..

[32] Li W., Wang Y., Ren H., Guo Z., Li N., Zhao C., Xie Z. (2023). Transcriptomic and physiological analyses identifying Lanzhou lily (*Lilium davidii* var. *unicolor*) drought adaptation strategies. Hortic. Plant J..

[33] Li W., Wang Y., Zhang Y., Wang R., Guo Z., Xie Z. (2020). Impacts of drought stress on the morphology, physiology, and sugar content of Lanzhou lily (*Lilium davidii* var. *unicolor*). Acta Physiol. Plant..

[34] Yin H., Chen Q., Yi M. (2007). Effects of short-term heat stress on oxidative damage and responses of antioxidant system in *Lilium longiflorum*. Plant Growth Regul..

[35] Gong B., Yi J., Wu J., Sui J., Khan M.A., Wu Z., Zhong X., Seng S., He J., Yi M. (2014). LlHSFA1, a novel heat stress transcription factor in lily (*Lilium longiflorum*), can interact with LlHSFA2 and enhance the thermotolerance of transgenic *Arabidopsis thaliana*. Plant Cell Rep..

[36] Tang Y., Tan B., Liu H., Liu Y., Zhang L., Zhang P., Sun M. (2026). A comprehensive review of physiological and molecular responses to stress of lilies (genus *Lilium*). Hortic. Res..

[37] Xu S., Chen R., Zhang X., Wu Y., Yang L., Sun Z., Zhu Z., Song A., Wu Z., Li T. (2024). The evolutionary tale of lilies: Giant genomes derived from transposon insertions and polyploidization. Innovation.

[38] Sun J., Wang X., Wang K., Meng D., Mu Y., Zhang L., Wang J., Yao G., Guo L. (2025). Genomic and epigenomic insight into giga-chromosome architecture and adaptive evolution of royal lily (*Lilium regale*). Nat. Commun..

[39] Arora R., Agarwal P., Ray S., Singh A.K., Singh V.P., Tyagi A.K., Kapoor S. (2007). MADS-box gene family in rice: Genome-wide identification, organization and expression profiling during reproductive development and stress. BMC Genom..

[40] Zhao Y., Li X., Chen W., Peng X., Cheng X., Zhu S., Cheng B. (2011). Whole-genome survey and characterization of MADS-box gene family in maize and sorghum. Plant Cell Tissue Organ. Cult..

[41] De Bodt S., Raes J., Van de Peer Y., Theissen G. (2003). And then there were many: MADS goes genomic. Trends Plant Sci..

[42] Maere S., De Bodt S., Raes J., Casneuf T., Van Montagu M., Kuiper M., Van de Peer Y. (2005). Modeling gene and genome duplications in eukaryotes. Proc. Natl. Acad. Sci. USA.

[43] Portereiko M., Lloyd A., Steffen J., Punwani J., Otsuga D., Drews G. (2006). *AGL80* is required for central cell and endosperm development in *Arabidopsis*. Plant Cell.

[44] Colombo M., Masiero S., Vanzulli S., Lardelli P., Kater M.M., Colombo L. (2008). *AGL23*, a type I MADS-box gene that controls female gametophyte and embryo development in *Arabidopsis*. Plant J..

[45] Kang I., Steffen J., Portereiko M., Lloyd A., Drews G. (2008). The AGL62 MADS domain protein regulates cellularization during endosperm development in *Arabidopsis*. Plant Cell.

[46] Steffen J., Kang I., Portereiko M., Lloyd A., Drews G. (2008). AGL61 interacts with AGL80 and is required for central cell development in *Arabidopsis*. Plant Physiol..

[47] Vining K., Romanel E., Jones R., Klocko A., Alves-Ferreira M., Hefer C., Amarasinghe V., Dharmawardhana P., Naithani S., Ranik M. (2015). The floral transcriptome of *Eucalyptus grandis*. New Phytol..

[48] Myburg A.A., Grattapaglia D., Tuskan G.A., Hellsten U., Hayes R.D., Grimwood J., Jenkins J., Lindquist E., Tice H., Bauer D. (2014). The genome of *Eucalyptus grandis*. Nature.

[49] Chen J., Yang Y., Li C., Chen Q., Liu S., Qin B. (2023). Genome-Wide Identification of MADS-Box Genes in *Taraxacum kok-saghyz* and *Taraxacum mongolicum*: Evolutionary Mechanisms, Conserved Functions and New Functions Related to Natural Rubber Yield Formation. Int. J. Mol. Sci..

[50] Xiong W., Risse J., Berke L., Zhao T., van de Geest H., Oplaat C., Busscher M., Ferreira de Carvalho J., van der Meer I.M., Verhoeven K.J.F. (2023). Phylogenomic analysis provides insights into MADS-box and TCP gene diversification and floral development of the Asteraceae, supported by de novo genome and transcriptome sequences from dandelion (*Taraxacum officinale*). Front. Plant Sci..

[51] Qiao X., Li Q., Yin H., Qi K., Li L., Wang R., Zhang S., Paterson A.H. (2019). Gene duplication and evolution in recurring polyploidization-diploidization cycles in plants. Genome Biol..

[52] Cui L., Cheng H., Yang Z., Xia C., Zhang L., Kong X. (2023). Comparative Analysis Reveals Different Evolutionary Fates and Biological Functions in Wheat Duplicated Genes (*Triticum aestivum* L.). Plants.

[53] Lawton-Rauh A. (2003). Evolutionary dynamics of duplicated genes in plants. Mol. Phylogenet. Evol..

[54] Luo X., He Y. (2020). Experiencing winter for spring flowering: A molecular epigenetic perspective on vernalization. J. Integr. Plant Biol..

[55] Pan W., Li J., Du Y., Zhao Y., Xin Y., Wang S., Liu C., Lin Z., Fang S., Yang Y. (2023). Epigenetic silencing of callose synthase by VIL1 promotes bud-growth transition in lily bulbs. Nat. Plants.

[56] Gao H., Wang Z., Li S., Hou M., Zhou Y., Zhao Y., Li G., Zhao H., Ma H. (2018). Genome-wide survey of potato MADS-box genes reveals that StMADS1 and StMADS13 are putative downstream targets of tuberigen StSP6A. BMC Genom..

[57] Ma H. (2005). Molecular genetic analyses of microsporogenesis and microgametogenesis in flowering plants. Annu. Rev. Plant Biol..

[58] Dreni L., Ferrándiz C. (2022). Tracing the Evolution of the *SEPALLATA* Subfamily across Angiosperms Associated with Neo- and Sub-Functionalization for Reproductive and Agronomically Relevant Traits. Plants.

[59] Zhao P.X., Miao Z.Q., Zhang J., Chen S.Y., Liu Q.Q., Xiang C.B. (2020). *Arabidopsis* MADS-box factor AGL16 negatively regulates drought resistance via stomatal density and stomatal movement. J. Exp. Bot..

[60] Chen R., Ma J., Luo D., Hou X., Ma F., Zhang Y., Meng Y., Zhang H., Guo W. (2019). CaMADS, a MADS-box transcription factor from pepper, plays an important role in the response to cold, salt, and osmotic stress. Plant Sci..

[61] Finn R.D., Clements J., Eddy S.R. (2011). HMMER web server: Interactive sequence similarity searching. Nucleic Acids Res..

[62] Finn R.D., Bateman A., Clements J., Coggill P., Eberhardt R.Y., Eddy S.R., Heger A., Hetherington K., Holm L., Mistry J. (2013). Pfam: The protein families database. Nucleic Acids Res..

[63] Chen C., Chen H., Zhang Y., Thomas H.R., Frank M.H., He Y., Xia R. (2020). TBtools: An Integrative Toolkit Developed for Interactive Analyses of Big Biological Data. Mol. Plant.

[64] Qi N., Hou X., Wang C., Li C., Huang D., Li Y., Wang N., Liao W. (2021). Methane-rich water induces bulblet formation of scale cuttings in *Lilium davidii* var. *unicolor* by regulating the signal transduction of phytohormones and their levels. Physiol. Plant.

